# Femoral head-neck junction reconstruction, after iatrogenic bone resection

**DOI:** 10.1093/jhps/hnv016

**Published:** 2015-02-03

**Authors:** Alberto Guevara-Alvarez, Nicholas Lash, Martin Beck

**Affiliations:** 1. Star Medica Hospital, Querétaro, México. Address: Blvd. Bernardo Quintana 4060, Col San Pablo, 76125, Querétaro, México; 2. Clinic for Orthopaedic and Trauma Surgery, Luzerner Kantonsspital, Luzern, Switzerland. Address: Spitalstrasse 16, 6000 Luzern, Switzerland; 3. Clinic for Orthopaedic and Trauma Surgery, Luzerner Kantonsspital, Luzern, Switzerland. Address: Spitalstrasse 16, 6000 Luzern, Switzerland

## Abstract

Arthroscopic over-resection of the head-neck junction during the treatment of a cam deformity can be a devastating complication and is difficult to treat. Large defects of the femoral head-neck junction (FHNJ) increase the risk of femoral neck fracture and can also affect hip biomechanics. We describe a case of an iatrogenic defect of the FHNJ due to excessive bone resection, and a previously non-described treatment using iliac crest autograft to restore femoral head-neck sphericity and hip joint stability. After protecting the femoral neck with an angled blade plate, the large anterior FHNJ defect was reconstructed using autogenous iliac crest bone graft; sphericity was restored by contouring the graft using spherical templates. Clinical and radiographic follow-up was performed up to 2 years. Results at 2 years showed no residual groin pain and normal range of motion. The Oxford Hip Score was 46/48, rated as excellent. Computed tomography (CT) scanning showed union of bone graft without resorption, and CT arthrogram indicating retained sphericity of the FHNJ without evidence of degenerative changes in the articular surface. This novel surgical technique can be used to restore the structural integrity and contour of the FHNJ that contains a significant anterior defect.

## INTRODUCTION

The femoral head-neck junction (FHNJ) plays an important role in the pathogenesis of femoro-acetabular impingement and resection of a cam deformity is the recommended treatment [1–6] There are several publications regarding the technique, amount of bone resection and possible complications of FHNJ resection [7–11]; however, the FHNJ can be a target for iatrogenic over-resection. Depending of the amount of resection the defect can lead to loss of labral seal and also stability of the hip.

To our knowledge there is no literature about reconstruction of the FHNJ after iatrogenic over-resection. Also the incidence of over-resection of a cam deformity is not known. However, a recent study showed that corrections at the head-neck-junction achieved by hip arthroscopy showed some overcorrection when compared with a surgical hip dislocation [12]. With increasing number of hip arthroscopies involving cam resection it has to be expected that such over-resections occur more frequently and that techniques of how to reconstruct the FHNJ may be needed. This technical note describes the treatment of a iatrogenic defect of the FHNJ due to surgical bone resection of incorrectly identified anatomy after treatment of a proximal femur fracture, and a novel treatment using iliac crest autograft to restore femoral head-neck sphericity.

## Case presentation

**Fig. 1.**
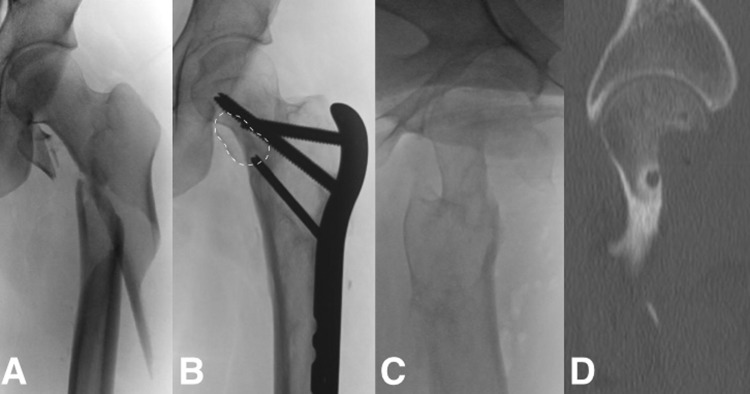
Initial presentation, subtrochanteric fracture with lesser trochanter avulsion **(A)**. Adequate consolidation of the subtrochanteric fracture with evident medial displacement of the lesser trochanter (dotted line) **(B)**, evident femoral neck junction resection after plate removal **(C)**, resection close to 50% confirmed by CT **(D)**.

A 17-year-old female pedestrian was struck by a motor vehicle sustaining a comminuted subtrochanteric femoral fracture (AO 32A1.1) (Fig. 1A). The patient underwent treatment of the fracture with open reduction and internal fixation trough a lateral approach with a proximal femoral locking plate. The main fragments of the fracture achieved union; however, the lesser trochanter remained displaced (Fig. 1B). At 3-month follow-up the patient complained of groin pain attributed to the displaced lesser trochanter and lateral thigh pain due to trochanteric bursitis and implant prominence. At that time the treating surgeon decided to remove the implant and resect the lesser trochanter. During surgery through a lateral approach, bone that was thought to be the lesser trochanter was resected. However, intraoperative documentation by fluoroscopy showed that the FHNJ inadvertently had been resected. The wound was closed and the case referred to the senior author.

Radiographic workup including standard radiographs, computed tomography (CT) and magnetic resonance imaging (MRI) was performed. This showed that 50% of the original cross-section of the FHNJ had been resected, posing a considerable fracture risk (Fig. 1C and D). MRI showed viability of the femoral head [13–15] and a reconstruction of the FHNJ together with prophylactic stabilization of the neck was planned.

The patient underwent surgery 3 weeks following the resection of the FHNJ. The patient was placed in lateral decubitus position and access was gained using a trochanteric flip osteotomy approach [8, 16]. A 20-cm long incision, including the previous incision was centered over the greater trochanter. The interval between gluteus medius and maximus was identified. A step cut osteotomy of the greater trochanter was performed and the interval between *piriformis muscle* and gluteus minimus developed. The greater trochanter was mobilized anteriorly and the joint capsule was identified. A Z-shaped capsulotomy was performed and the hip joint was inspected but not dislocated. The bone defect started at the equator of the femoral head and included ∼50% of the neck diameter and extended distally to the intertrochanteric line (Fig. 1D, 2A). The lateral retinaculum with the terminal branches of the deep branch of the medial femoral circumflex artery was intact. Under direct vision and image intensifier, a 130°/4.5-mm angled blade plate was inserted. The hip was then dislocated after release of the ligamentum capitis femoris. Viability of the femoral head was proven by bleeding evident from a drill hole done in the femoral head fovea [17]. The weight bearing cartilage of both the femoral head and acetabulum, and chondrolabral complex was undamaged.

**Fig. 2.**
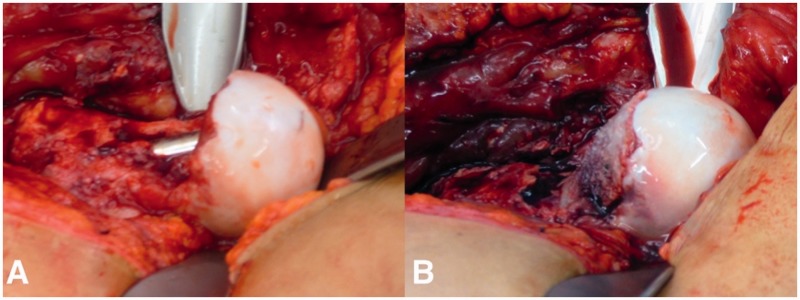
Intraoperative view of the dislocated hip after stabilization with the angled blade plate. The defect of the FHNJ is visible **(A)**. View after reconstruction with cancellous bone graft **(B)**.

Bi-cortical iliac crest graft was harvested through a separate incision and contoured to fit the defect. It would have been preferable to place the cancellous side against the bone of the defect and the smooth cortical surface facing the joint. However, the curvature of the cortical surface of the graft was concave and it was not feasible to contour it with the cortical surface facing the joint. Therefore, the cortex was removed and the graft was pre-contoured and fixed into the defect with two 2.0-mm titanium screws. The FHNJ was then contoured using a burr which corroborated with a 42-mm spherical template to restore sphericity (Fig. 2A and B).

**Fig. 3.**
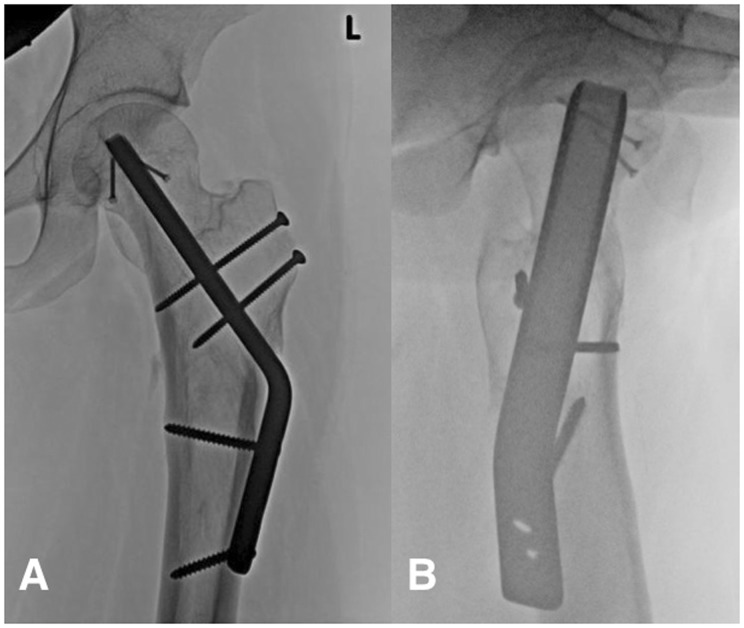
Two-year postoperative AP radiograph **(A)** and lateral **(B)** showing adequate fixation and consolidation of the trochanteric osteotomy without changes in the femoro-acetabular joint.

A gap of 1 mm remained between the reconstructed femoral head and the spherical template to allow room for synovial membrane to envelope the reconstructed cancellous surface. The hip was then reduced and range of motion tested to ensure it remained impingement free. The capsule was sutured loosely and the trochanteric osteotomy fixed with two 3.5-mm cortical screws. Continuous passive motion was started the first day after surgery and the patient was discharged, partial weight bearing for 6 weeks, full weight bearing thereafter. Clinical and radiographic follow up was performed at 6 and 12 weeks, sixth month and at 1 and 2 years. (Fig. 3A and B).

**Fig. 4.**
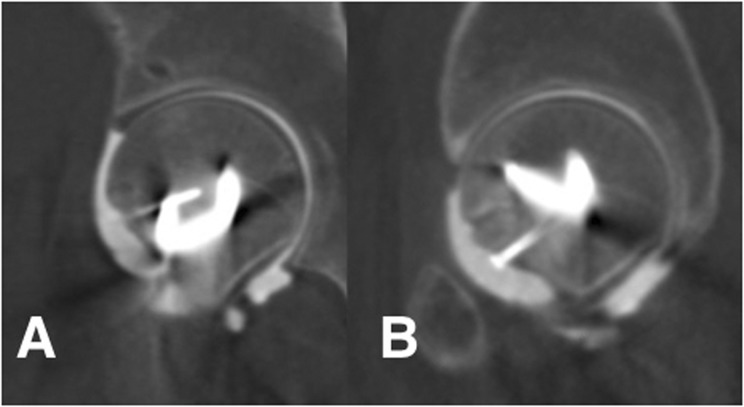
CT arthrogram showing site of graft with full consolidation and no residual defect **(A)** as well as adequate sphericity in the most anterior part of the FHNJ in a sagittal view **(B)**.

At 2-year follow-up the patient had no pain or signs or symptoms of instability of the hip. There was some tenderness over the proximal aspect of the plate. The Oxford hip score was 46/48 points, rating as excellent. The range of motion of the left hip has a coxo-femoral flexion of 100°; extension 10°; internal rotation of 30°; external rotation 60°; abduction of 40°. Impingement sign was negative. Abductor strength was rated 5/5. There were no radiographic signs of osteonecrosis. CT showed the bone graft was fully integrated and CT-arthrogram coronal (Fig. 4A) and oblique (Fig. 4B) slides showed restored sphericity of the FHNJ.

## DISCUSSION

The incidence of over-resection of the FHNJ is unknown, but it was shown recently that corrections at the head-neck-junction achieved by hip arthroscopy showed some overcorrection when compared with open resection via a surgical hip dislocation [12]. Also, it was shown that assessment of correct resection is difficult at the time of surgery [6, 9, 18]. When over-resection is present it poses a problem with potentially catastrophic consequences and difficult to treat. We present a previously undescribed technique which may help surgeons to address this important problem.

When faced with a large femoral head-neck defect there are two issues to be dealt with, loss of sphericity and structural weakness/fracture risk.

Ferguson *et al*. [16], has shown that a spherical femoral head is needed to support normal labral function in creating the negative pressure suction effect, imparting hip stability and lubrication. This is lost with large femoral head-neck defects. Additionally, significant anterior defects the femoral head may allow for the head to translate in flexion leading to instability.

Mardones *et al*. [9] and Rothenfluh *et al*. [19] have both shown that fracture risk directly correlates with depth of resection. Resections of between 10 and 30% can increase fracture risk and the length and width can also impact on it. In a finite element study a depth of resection is suggested to be limited to 10 mm [20].

There are three things the surgeon should be aiming to achieve: (i) recreation of sphericity of the head, (ii) restoration of bone stock for strength and (iii) protection of the neck from fracture whilst the graft incorporates. The prophylactic fixation is performed first to allow safe dislocation of the hip and can be done with any angular stable device. The recreation of the femoral head with autograft affords immediate return of spherical anatomy with excellent incorporation potential, but also overtime restoration of bone strength is imparted, allowing implant removal once united. The use of the flip osteotomy allows excellent visualization of the defect and surrounding anatomy to allow accurate reconstruction.

This article presents a novel technique in case of over-resection of the FHNJ, which allows the surgeon to recreate the normal anatomy, as well as the hip function, diminishing the risk of fracture, preserving the original bone, without considering a total hip arthroplasty as an alternative, in this young patients, where joint preserving surgery was the primary aim of the treatment.

## ETHICAL BOARD REVIEW STATEMENT

The authors certify that his or her institution has approved the human protocol for this investigation and that all investigations were conducted in conformity with ethical principles of research.
